# Hazard proximity and risk perception of tsunamis in coastal cities: Are people able to identify their risk?

**DOI:** 10.1371/journal.pone.0186455

**Published:** 2017-10-31

**Authors:** Juan Pablo Arias, Nicolás C. Bronfman, Pamela C. Cisternas, Paula B. Repetto

**Affiliations:** 1 Engineering Sciences Department, Universidad Andres Bello, Santiago, Chile; 2 National Research Center for Integrated Natural Disaster Management CONICYT/FONDAP/15110017, Santiago, Chile; 3 Department of Psychology, Pontificia Universidad Católica de Chile, Santiago, Chile; Albert-Ludwigs-Universitat Freiburg, GERMANY

## Abstract

Researchers have previously reported that hazard proximity can influence risk perception among individuals exposed to potential hazards. Understanding this relationship among coastline communities at risk of flood events caused by storms and/or tsunamis, is important because hazard proximity, should be recognized when planning and implementing preparation and mitigation actions against these events. Yet, we are not aware of studies that have examined this relationship among coastline inhabitants facing the risk of a tsunami. Consequently, the aim of this study was to examine the relationship between hazard proximity and perceived risk from tsunamis among coastline inhabitants. Participants were 487 residents of the coastal city of Iquique, Chile. They completed a survey during the spring of 2013 that assessed their perceived risk from several natural and non-natural hazards. We found that hazard proximity maintains a negative relationship with the perception of tsunami risk among coastline inhabitants. While this result confirms the general trend obtained in previous studies, this one is conclusive and significant. In contradiction with previous findings, we found that participants from the highest socioeconomic status reported the highest levels of risk perception. This finding can be explained by the fact that most participants from the highest socioeconomic status live closer to the coastline areas, so their risk perception reflects the place where they live, that is in a tsunami inundation zone. Once again, hazard proximity proved to be a determinant factor of risk perception. Our findings have important implications for the development of plans and programs for tsunami preparedness and mitigation. These indicate that individuals do use environmental cues to evaluate their own risk and can potentially make correct choices when having or not to evacuate. Also suggest that preparedness should incorporate how hazard proximity is recognized by individuals and communities at risk.

## Introduction

Earthquakes and tsunamis impact coastline communities around the globe, causing many casualties and incalculable economic losses. In the last two decades alone, more than 750,000 people lost their lives because of an earthquake or tsunami, and economic losses were close to 787 trillion US dollars [1]. Additionally, it is estimated that 13% of the urban population lives 10 meters or less above sea level, a number that it is expected to continue growing in the coming decades [2].

This scenario makes urgent to prepare coastal communities to cope with these hazards in a timely and effective manner. Several authors have suggested that the motivation and willingness to take preventive actions are directly related to individual levels of risk perception [3–5]. Overall, they have concluded that the higher the perceived risk, the higher the probability that the individual is willing to take preventive actions to mitigate the associated risks [6, 7]. Still, it has also been found that, in some cases, a higher perception of risk does not necessarily imply a greater willingness to take preparedness and mitigation actions against the occurrence of natural hazards [8, 9]. This complex relationship reveals the importance to evaluate the role of other factors to further explain risk perception.

### Risk perception and hazard proximity

Environmental cues are recognized among the factors that influence risk perception, showing that individuals incorporate their observations to assess their own risk [10]. In fact, proximity to technological hazards have been found to significantly influence perceived risk among individuals who face these hazards [9, 11–13]. Early studies exploring this relationship on technological risks, such as nuclear power plants [14], chemical plants [15], and transportation of hazardous substances [16], among others, have concluded that the closer to the hazard source the higher the perceived risk [17].

Recently, the relationship between hazard proximity and risk perception has interested researchers who have studied its role in relation to some natural hazards, mainly flooding riverbeds [18], coastline floods [19], volcanoes [20] and earthquakes [21]. However, their findings have not been consistent, while some researchers have found a positive relationship [20, 22], others have failed to do so [23]. Lindell and Perry [7] argue that these contradictory findings reveal that in some scenarios people have limited ability to identify their location in hazardous areas, while in others they may have greater ability. For example, Arlikatti et al. [23] examined the accuracy with which 359 residents of the United States´ west coast were able to locate their residence in risk maps for hurricanes. They found that only one third of the participants correctly identified their risk areas given their residential location.

Barberi et al., [20] evaluated risk perception among individuals living close to the Vesuvius volcano in Italy. The sample of 2,488 participants were divided into red and yellow zones, according to their hazard proximity. Participants living in the red zone reported greater risk perceptions as compared to those in the yellow zone. In other study, Lindell and Hwang [22] examined the relationship between hazard proximity, hazard experience and perceived personal risk for three hazards: flooding from rivers, hurricanes and chemical plants in a sample of 321 participants living in the United States. They found that risk perception was correlated with coastline (risk of hurricanes) and chemical plant proximity, but not with river proximity (flood risk). Their results provide evidence of a direct effect of proximity hazard on the risk perception for hurricanes and chemical hazards, but not for river flooding, and suggest that the nature of the hazard plays an important role when examining this relationship.

Similarly, Zhang et al., [24], evaluated how the housing price is affected by the relationship between hazard proximity and risk perception when facing natural and technological hazards, in a sample of 200 households in the United States. The correlation analyses revealed that the relationship between hazard proximity and risk perception regarding floods, hurricanes and chemical hazards is negative and statistically significant. Bubeck et al. [25], in a review of studies evaluating risk perception and precautionary behavior found that, in general, the distance to a river or a body of water exerts a small effect on current mitigation measures (such as flood insurance) adopted by people. These differences in findings suggest that the relationship between hazard proximity and risk perception may not be the same for all hazards, as has been proposed by Lindell and Perry [7]. The ability to recognize the threat may differ according to the nature of the hazard and maybe relevant to explore. The study of other natural hazards may help to better understand this relationship.

Despite the increased interest in studying the relationship between perceived risk and hazard proximity to natural disasters (mainly flooding due to weather events), to this date, we have not found studies examining this relationship among coastline inhabitants who face the risk of a tsunami. Studies in this area are essential because we know substantially less from tsunamis than others natural hazards such floods or hurricanes, and the fact that the location of the hazard may be easier to identify, may contribute to our understanding of the role of hazard proximity and risk perception [26]. Moreover, study whether, population living in risk zones for tsunamis in locations where the source of the tsunami that is close to the land and the time to issue an official warning is limited, recognize proximity of the hazard in their perception of risk, may contribute to inform and develop more effective preparedness actions. For example, inhabitants of coastal communities who were exposed to the 2015 Illapel earthquake and tsunami reported that the waves arrived between 2 [27] and 12 minutes after the earthquake [28], giving very limited time for the official warning to be issued. Fortunately, no victims were reported: inhabitants evacuated voluntary and promptly after the earthquake. They rapidly recognized they were on a risk zone and needed to reach safety.

In fact, the hazard zone for a tsunami may be easier to identify as compared to other hazards involving bodies of water, however this is something that has not been addressed previously. This is the main gap we intend to address with the present study. Findings from this study could contribute to better understand the relationship between hazard proximity and risk perception. Consequently, the main objective of this study was to examine the relationship between hazard proximity and the perceived risk for tsunami among coastline residents. In line with previous studies, we expected residential location to be a determining factor of the public perception of risk from a tsunami; that is, that hazard proximity relates inversely and significantly with risk perception.

#### Risk perception and sociodemographic factors

The relationship between risk perception and sociodemographic factors such as age, gender and socioeconomic status has been widely studied in the literature [29, 30]. In general, it has been found that women report greater perceived risk from natural hazards such as earthquakes [31], volcanoes [20] and floods [19]. With respect to age, the literature shows greater perception of risk from natural hazards among older people [3, 32]. Regarding socioeconomic status, studies show that as the status of people increases, their perception of risk from natural hazards decreases [22, 24]. These factors will be included as covariates in the present study.

### Chile and its coastline: The case study

**Fig 1:**
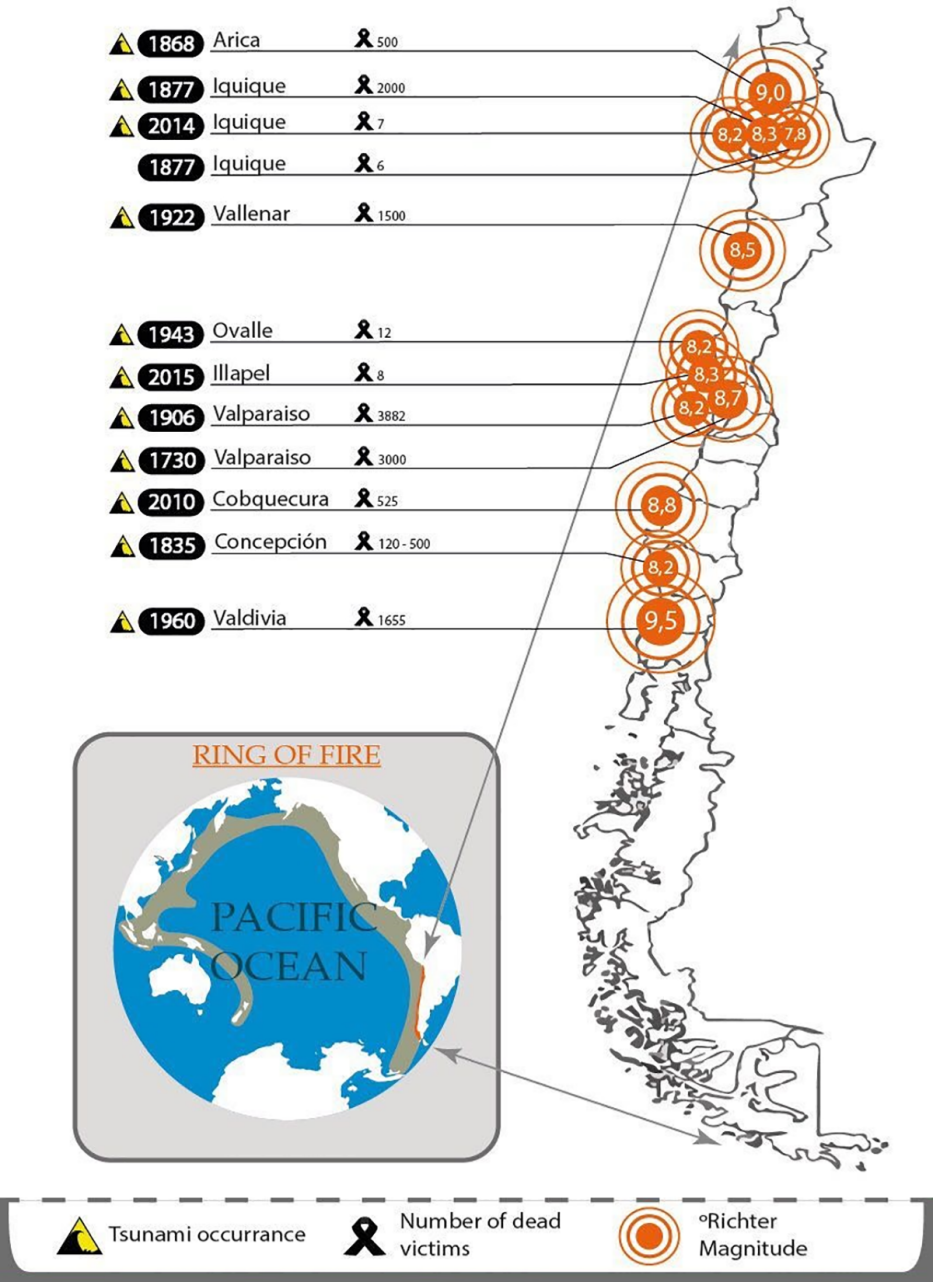
Map of Chile. Twelve of the most destructive earthquakes in the history of Chile.

Chile is a narrow strip of land between the Andes and the southeastern coast of the Pacific Ocean (see Fig 1). It comprises a length of 4,270 km. reaching a maximum width of 445 km. Chile is located in the Pacific Ring of Fire and is exposed to one of the highest frequency of occurrence of earthquakes and tsunamis. In the XXI century alone, 79 earthquakes of a magnitude ≥7.0 on the Richter scale have occurred in Chile [33]. In fact, the largest earthquake ever recorded in history took place in the city of Valdivia in 1960, with a magnitude of 9.5 on the Richter scale (see Fig 1). The mega-event was due to a rupture of 1,000 km. in length, which significantly altered the geography of the affected area and triggered a destructive tsunami, with waves ranging between 10 and 15 meters [34], which reached the coasts of Japan, the United States and Hawaii, among other places. In Chile, the effect was devastating. In 1960 the country's population was just over 7.3 million inhabitants [35]. This earthquake killed more than 2,000 people, affected about 2 million people and caused economic losses of more than 550 million dollars [36].

The coastline cities of Chile most frequently affected by earthquakes and/or tsunamis are in the northern and central region. Iquique, located in the north of the Chilean coast is one of these cities (see Fig 1). The largest seismic event recorded in the area corresponds to a megathrust earthquake of 8.8 magnitude on Richter scale, which occurred in 1877 and generated a tsunami with waves between 9 and 14 meters high [37]. Because of insufficient seismic activity, the scientific community has predicted the occurrence of a seismic event of great magnitude in the subduction zone adjacent to northern Chile [38] and expect that this may occur sometime soon.

In fact, on April 2014, an earthquake of an 8.2 magnitude on Richter scale shook the city of Iquique. The event killed six people and more than 13,000 homes were affected. Economic losses were estimated at 100 million USD. While at one point, the public thought it was the great earthquake foreshadowed by the scientific community, researchers, however suggested the opposite. In fact, Hayes and colleagues concluded that "…Significant sections of the northern Chile subduction zone have not ruptured in almost 150 years, so it is likely that future megathrust earthquakes will occur to the south and potentially to the north of the 2014 Iquique sequence." [39]. Therefore, the community of Iquique is still waiting for this event to occur.

Considering this scenario and aiming to contribute to design and implement plans and programs to prepare coastal communities in Chile to cope effectively with natural hazards, we studied the inhabitants of the city of Iquique.

## Methods

We used data from the survey study conducted by Bronfman et al. [40], in 2013 (July to October) that aimed to evaluate risk perceptions in the Chilean population and the degree of trust they have in institutions in charge for preparedness, response and recovery in the context of natural disasters. This study was implemented in five major cities in Chile: Iquique, Antofagasta, Valparaíso, Santiago and Concepción. The sample was stratified by gender, socioeconomic status and age, and was representative of each five cities. A total of 2,054 face-to-face interviews were held in the homes of each participant.

The questionnaire included three sections. The first section measured risk perception and the degree of acceptability regarding 40 hazards grouped into eight categories: (1) five transportation hazards, (2) four forbidden or addictive substances, (3) four social ills, (4) four environmental hazards, (5) four chemical products and substances, (6) six technological hazards, (7) nine natural hazards and (8) four other hazards. The risk perception measure used was designed based on previous studies that have addressed similar problems [22, 41–44], within the psychometric tradition [45]. The following question was used to assess directly the perception of risk of each hazard: *how much risk do you think the national population is (will be) exposed as consequence of a (**place the hazard here*)? Participants had to respond using a 7-point bipolar scale from (1) “no risk” to (7) “high risk”.

Section 2 assessed social trust in ten Chilean institutions who are in charge for preparedness, response and recovery from the occurrence of a natural disaster. Finally, section 3 included sociodemographic questions for participants. Further details on the survey and its results can be found in Bronfman et al. [40]. The Ethics Committees of the Funding Institution (CONICYT) and the one in University Andrés Bello approved all procedures.

### Participants and measures

For the purposes of this study we used data collected in Iquique about risk perception of earthquakes and tsunamis in Bronfman et al., [40] study (S1 Dataset). The sample included 487 adults and 49.7% were females. Mean age was 45 years. Sample distribution by age group was: 24.9% (18–29 years), 23.9% (30–44 years), 25.5% (45–59 years) and 25.7% (60 years).

The original questionnaire included 40 hazards, nine of which were natural hazards. For the purposes of the present study we are only interested in measuring tsunami risk perception. Since the Chilean coastal population is well aware that the occurrence of a tsunami is linked to the occurrence of an earthquake near the coast [46, 47], we used the mean value between risk perception for earthquakes and risk perception for tsunamis as a general measure of risk perception for each participant (reliability of scale: *α* = 0.86). We also included all questions that evaluated socioeconomic and demographic conditions of the participants.

**Fig 2:**
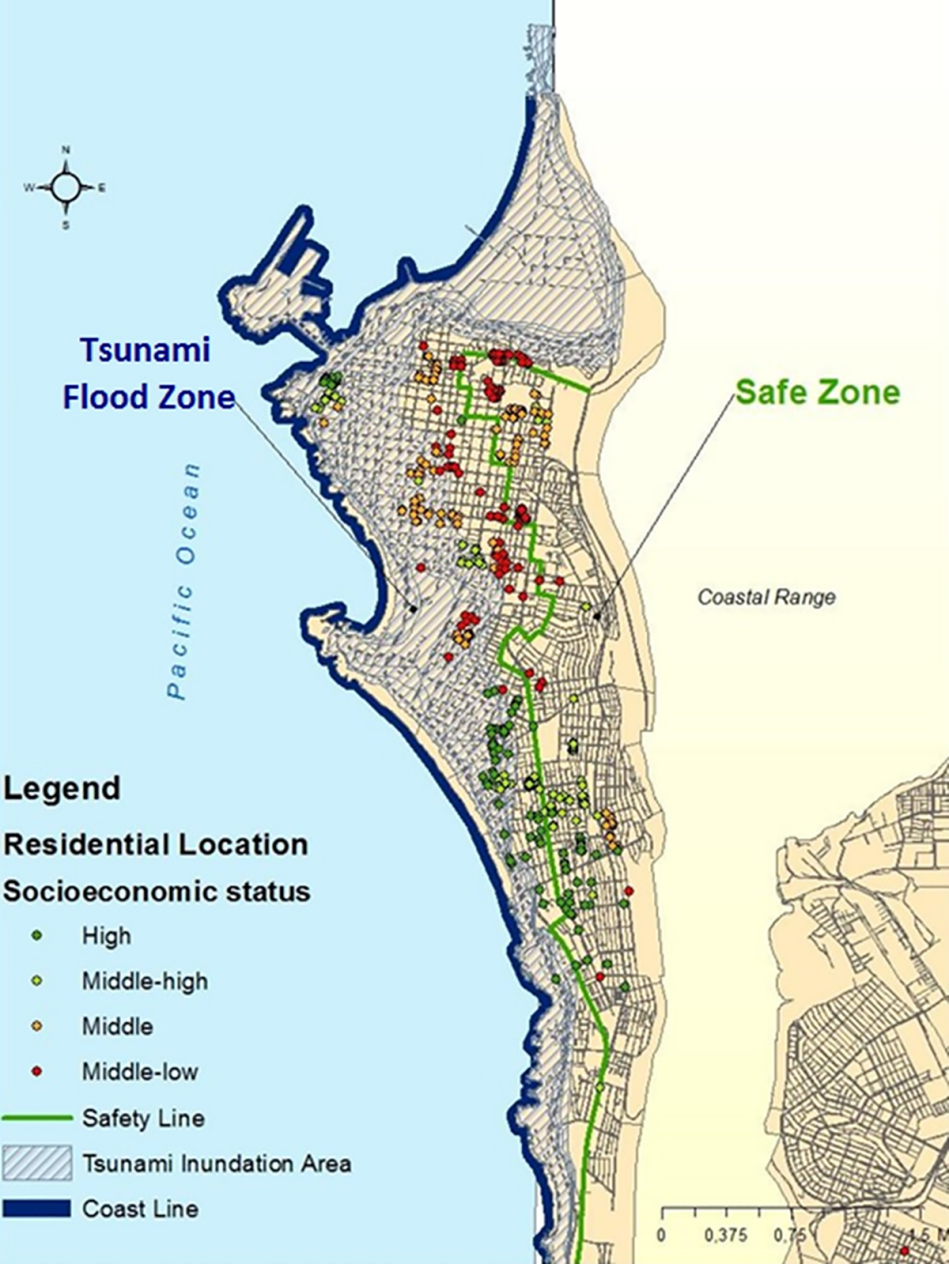
Residential location of the survey’s participants by household socioeconomic status. Based on data from the 2002 Census of Chile data layer. (1) High, (2) Middle-High, (3) Middle and (4) Middle-Low. The map was elaborated by the authors and it is not subject to any copyright restriction.

To determine whether each participant lived in a risk zone or not, we used the residential location information for each respondent that was collected with the survey. This information allowed us to locate the geographic coordinates (latitude/longitude), and then, using ArcGIS and the 2002 Census of Chile data layer we geospatially located each participant’s home. Fig 2 shows the spatial distribution of participant’s homes within the zones of Iquique.

### Data analysis

To classify each participant´s household by risk (at risk or not for tsunami), we used government information that splits Iquique in two zones: *Safe Zone* and *Tsunami Flood Zone*. The boundary between the two areas, which we labeled *Safety Line*, was determined using the tsunami safety line defined by the National Emergency Office of the Interior Ministry of Chile [48]. This line is located 30 meters above sea level (masl), based on the historical analysis of the height reached by major tsunamis in the world conducted by the *International Tsunami Information Center*, *Hawaii* (ITIC). Historically the waves caused by a tsunami do not exceed 30 masl [49, 50].

To address our research question, we conducted several analyses. We first performed descriptive analyses and then compared risk perception among those participants in the safe zone with the ones in the tsunami flood zone. We also compared the risk perception based on demographics. All data was analyzed using SPSS, version 22.0.

## Results

Table 1 shows the mean values for perceived risk measure against an earthquake/tsunami with the sample stratified by residential location (safe area and tsunami flood zone) and according to sociodemographic variables. Our results suggest that, in general terms, the population living in the tsunami flood zone (below the level of 30 meters) reports a risk perception significantly higher *(p* <0.05) as compared to those residing in the safe zone. It is important to note, however that, overall the average score of risk perception in both areas is substantially high (5.65 and 5.96 on a 7-point scale).

**Table 1:** Mean values (and *SD*s) of risk perception earthquake/tsunami with sample stratified by socio-demographic variables and residential location (safe and tsunami flood zone).

	All Sample		Safe Zone		Tsunami Flood Zone		*p*-value^†^
**All sample**	5.85	*(1*.*57)*	5.65	*(1*.*63)*	5.96	*(1*.*53)*	0.036
**Gender**							
*Male*	5.85^a^	*(1*.*55)*	5.72^a^	*(1*.*53)*	5.93^a^	*(1*.*56)*	0.312
*Female*	5.85^a^	*(1*.*60)*	5.58^a^	*(1*.*73)*	6.00^a^	*(1*.*51)*	0.062
**Age group**							
*18–29*	5.55^a^	*(1*.*67)*	5.20^a^	*(1*.*72)*	5.81^a^	*(1*.*60)*	0.044
*30–44*	5.60^a^	*(1*.*69)*	5.23^a^	*(1*.*90)*	5.79^a^	*(1*.*56)*	0.116
*45–59*	6.04^ab^	*(1*.*51)*	6.05^ab^	*(1*.*36)*	6.04^a^	*(1*.*60)*	0.955
*60+*	6.18^b^	*(1*.*33)*	6.18^b^	*(1*.*22)*	6.17^a^	*(1*.*38)*	0.997
**Socioeconomic status**		** **					
*High*	6.31^a^	*(1*.*20)*	6.34^a^	*(1*.*12)*	6.29^a^	*(1*.*24)*	0.703
*Middle-high*	5.91^ab^	*(1*.*58)*	6.20^a^	*(1*.*23)*	5.77^a^	*(1*.*72)*	0.137
*Middle*	5.50^b^	*(1*.*62)*	5.08^b^	*(1*.*57)*	5.83^a^	*(1*.*59)*	0.007
*Middle-low*	5.73^b^	*(1*.*73)*	5.38^b^	*(1*.*98)*	5.93^a^	*(1*.*54)*	0.110

Reading by column for each socio-demographic variable, mean values with different letters are significantly different at the *p* < 0.01 level (Tukey's *HSD*).

^†^Statistical significance between the mean difference for safe and unsafe zones.

When comparing risk perception by sex, the results show no significant differences between males and females, regardless of their residential location. We did not find differences within sex groups in relation to their residential location.

Comparisons between age groups show that, in general, adults and seniors (45 years of age and older) declared significantly higher risk perception for earthquakes/tsunamis than younger age groups. The same trend was observed among participants residing in the flood zone, but the difference was not statistically significant. Similarly, when comparing different age groups according to their residential location, we found that younger adults (29 and younger) who live in the safe zone show a significantly lower risk perception *(p* <0.05) to those in the same age group living in the flood zone. For adults over 30, we found no significant differences in risk perception based on residential location.

Our results show that, overall, participants in the highest socioeconomic status reported higher levels of perceived risk. Meanwhile, participants of middle socioeconomic group, residing in safe zone declared a significantly lower risk perception *(p* <0.01) than their counterparts living in the flood zone. When analyzing the spatial distribution of participants based on their socioeconomic status, we found that homes of participants from higher socioeconomic levels are located at shorter distance from the coastline (27%) than the homes of participants from lower socioeconomic levels (24%) (See Fig 2).

**Fig 3:**
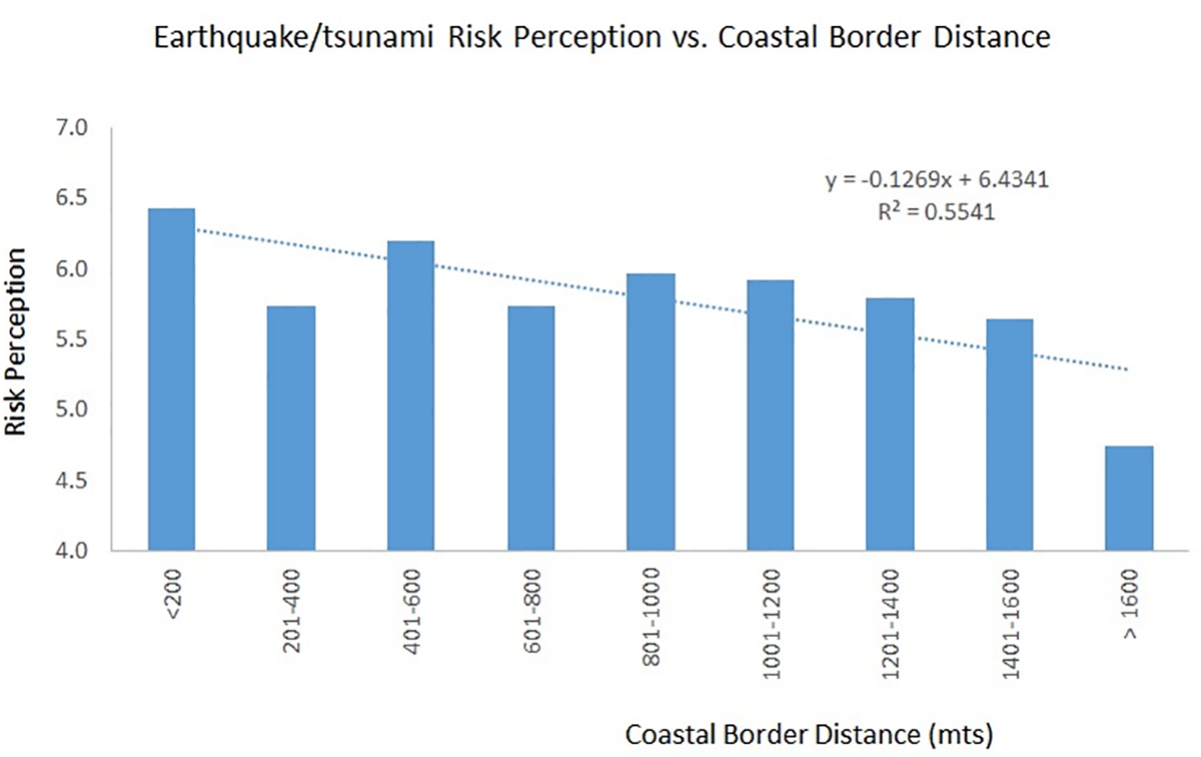
Earthquake/Tsunami risk perception versus coastal border distance. Relationship between earthquake/tsunami risk perception and hazard proximity.

The relationship between risk perception of an earthquake/tsunami and the proximity to the coastline is represented in Fig 3. This figure shows that the greater the distance between the participant´s residence (household) and the waterfront, the lower the perceived risk of an earthquake/tsunami is. That is, risk perception still has a negative and statistically significant relationship *(p* <0.05) with hazard proximity.

In order to further understand the relationship between risk perception, hazard proximity and socioeconomic status we conducted first-order partial correlation analysis. Correlations were conducted with each pair of variables and keeping the remaining as constant. Table 2 shows *Pearson’s* correlations and partial correlations obtained for each pair of variables. The results show that the linear relationship between hazard proximity and risk perception keeps the direction and the statistical significance (*p* < 0.05) when the correlation is controlled by the socioeconomic status. Thus, we can conclude that the relationship between risk perception and hazard proximity is not attributable to socioeconomic status.

**Table 2:** Pearson correlation and first-order partial correlations between perceived risk, hazard proximity and socioeconomic level.

	Pearson Correlations		Partial Correlations	
	HP	SES	HP	SES
**RP**	-0.147**	-0.155**	-0.091*	-0.103*
**SES**	0.421**	1.000	0.411**	1.000

RP = Risk Perception; HP = Hazard Proximity; SES = Socioeconomic Status.

*p < 0.05

** *p* < 0.01.

## Discussion

Our main results are consistent with our initial hypothesis, that is, hazard proximity relation on the perception of risk for earthquakes/tsunamis for inhabitants of the coastline remains negative and statistically significant. Thus, shorter distances from the coastline result in higher risk perception. This result confirms the general trend obtained in previous studies that have examined this relationship [9, 18–20, 22, 24].

These conclusive results may be explained by the greater ability of the inhabitants studied to recognize their proximity to the hazard, as compared to participants in previous studies focusing on other natural hazards [7, 23]. Several factors may explain this greater hazard proximity recognition, such as visibility of hazard, previous experience and available information. People residing in the city of Iquique live, work and perform their daily activities on the coast. They have also experienced earthquakes and tsunamis previously. Their previous experience with earthquakes and tsunamis may facilitate their ability to recognize their proximity with this hazard. Also, the actions taken by local authorities and experts in order to keep the population aware and prepare, have probably contributed to their ability to recognize the hazard. This is a significant finding because it provides more evidence of the relevance of the nature of the hazard when studying the role of hazard proximity on risk perception, and contributes to understand the contradictory findings reported previously.

While this increased public concern about the risk of earthquakes and tsunamis would increase the willingness to take preventive actions to mitigate the associated risks [4], it could also may have potential negative consequences when a tsunami evacuation occurs [40]. Residents living in a safe area who have high risk perception could potentially evacuate to higher areas (shadow evacuation), blocking escape routes for those coming from flood risk areas [23]. This could be potentially catastrophic in situations where the evacuation is carried out mainly in cars, blocking evacuation routes and hindering the flow of people. Thus, our results reveal that the people’s recognition of the hazard proximity is crucial for the tsunami evacuation process and should be taken into account when planning and implementing tsunami evacuation plans and programs.

Previous studies have shown that women report greater risk perception of natural hazards as compared to men [19, 20, 31]. Unexpectedly, our results showed no significant differences between men and women, regardless of the area of residence of the participant. It is possible that the scale used was not able to capture these gender differences; possibly the measurement scale used was not wide enough to display these differences. In fact, the high scores reported in risk perception (at the upper limit of the scale) by both men and women in the present study could also have limited the possibility to detect gender differences. Future studies should further examine gender differences incorporating additional measures of risk perception.

Consistent with previous studies [3, 32], older participants living in safe areas reported higher risk perception. Additionally, we found that people 29 and under living in safe areas showed a significantly lower risk perception than people of the same age group living in a tsunami flood zone. This may also be explained by the effect of hazard proximity, but also may suggest that actions taken to prepare the communities may be reaching younger population. This is particularly concerning since older adults are considered vulnerable and present difficulties to successfully cope with disasters [51]. Actions aimed to target older adults to prepare them to effectively cope with a potential tsunami are necessary.

Surprisingly, participants from higher socioeconomic status groups reported overall higher risk perception as compared to residents of lower socioeconomic status. This result contradicts findings from previous studies [22, 24]. This result, however, reflects the place where these participants live. Most participants from the highest socioeconomic status live closer to the coastline areas, so their higher risk perception may reflect their actual location. Thus, hazard proximity proved to be a significant influential factor in risk perception, even changing the traditional difference found in previous studies for socioeconomic status. Nevertheless, to further understand this relationship, longitudinal studies should be conducted in order to examine how risk perception changes over time and the role that taking actions to prepare and mitigate for these hazards could have on the perceived risk [26].

Our results provide evidence of the need to consider the role of hazard proximity both for the study of risk perception as well for the preparation of communities exposed to natural hazards. It also recognizes that communities and individuals recognize these risks and use environmental information to effectively cope with natural hazards, such as earthquakes and tsunamis that cannot be accurately predicted. Our results also reveal that hazard proximity should be actively incorporated in the strategies aimed to prepared communities and in the development of risk communication plans, particularly in a tsunami scenario, where non-structural mitigation efforts are very relevant to prevent unnecessary casualties. They also suggest that preparation actions should build on what individuals and communities have learned from past events and explicitly identify the hazard zone and its nature. These actions should build from already implemented actions aimed to provide hazard zone maps to the public. Keeping the inhabitants of Iquique aware of their risk through several actions appear to have helped to maintain them alert and reporting high risk perception. Moreover, the differences between groups, should also be considered when targeting groups that are at risk because they are less likely to evacuate or potentially do it when it is not required, and groups that decide to evacuate when is not required.

## Limitations

Some limitations of the present study must be recognized. We assessed risk perception in one moment in time and therefore we cannot conclude there is a casual relationship between the two main study variables [26]. We also used a limited number of items that focused on the public perception of risks (10). However, the risk perception model used in this study has a long-standing tradition [43, 52–55]. This approach asks participants to evaluate risks from a long list of scenarios. In the present study, we use two questions to evaluate the social perception of risk, and ask participants directly to evaluate the national population risk as consequence of (i) an earthquake and (ii) a tsunami. Risk attributes as voluntariness, event frequency of occurrence, severity of the consequences, familiarity, social degree of knowledge, dread and catastrophic potential, among others, are important elements that influence the people’s risk judgments and they should be considered in future studies.

Our results, however, are consistent with findings from previous studies focusing on hazard proximity and risk perception, with studies have also measured risk perception with two items [10]. The fact that we used measures that have been previously by other researchers in the field and that most of our findings are consistent with previous studies, suggest that, despite these limitations, our findings are useful to further understand the role of hazard proximity in risk perception to natural hazards. Nevertheless, future studies could incorporate measures of risk perception that include other dimensions, could allow to contribute to better understand the role of hazard proximity in relation to risk perception.

Participants in the study were from a coastal city in Chile that may not be representative of other individuals exposed to similar hazards in other places around the world. The fact that the fault is near to the city and inhabitants have been exposed to these events previously makes them a particularly interesting group to study: they may be well-aware of the hazard and recognize the risk. Differences in findings can be explained by the type of hazard studied, particularly the fact that tsunami risk zone maybe easier to identify as compared to others, and this greater awareness [26].

## Supporting information

S1 Dataset Data set used in the research. (XLSX)
